# Microstructural and functional impairment of the basal ganglia in Wilson’s disease: a multimodal neuroimaging study

**DOI:** 10.3389/fnins.2023.1146644

**Published:** 2023-04-20

**Authors:** Dongning Su, Zhijin Zhang, Zhe Zhang, Yawen Gan, Yingkui Zhang, Xinyao Liu, Jingfeng Bi, Lingyan Ma, Huiqing Zhao, Xuemei Wang, Zhan Wang, Huizi Ma, Shairy Sifat, Junhong Zhou, Wei Li, Tao Wu, Jing Jing, Tao Feng

**Affiliations:** ^1^Department of Neurology, Beijing Tiantan Hospital, Capital Medical University, Beijing, China; ^2^China National Clinical Research Center for Neurological Diseases, Beijing, China; ^3^Tiantan Neuroimaging Center of Excellence, Beijing Tiantan Hospital, Capital Medical University, Beijing, China; ^4^Djavad Mowafaghian Centre for Brain Health, Pacific Parkinson’s Research Centre, University of British Columbia and Vancouver Coastal Health, Vancouver, BC, Canada; ^5^Hinda and Arthur Marcus Institute for Aging Research, Hebrew SeniorLife, Roslindale, MA, United States; ^6^Harvard Medical School, Boston, MA, United States

**Keywords:** Wilson’s disease, multimodal MRI, basal ganglia, iron, neuroimaging biomarker

## Abstract

**Objectives:**

Magnetic susceptibility changes in brain MRI of Wilson’s disease (WD) patients have been described in subcortical nuclei especially the basal ganglia. The objectives of this study were to investigate its relationship with other microstructural and functional alterations of the subcortical nuclei and the diagnostic utility of these MRI-related metrics.

**Methods:**

A total of 22 WD patients and 20 healthy controls (HCs) underwent 3.0T multimodal MRI scanning. Susceptibility, volume, diffusion microstructural indices and whole-brain functional connectivity of the putamen (PU), globus pallidus (GP), caudate nucleus (CN), and thalamus (TH) were analyzed. Receiver operating curve (ROC) was applied to evaluate the diagnostic value of the imaging data. Correlation analysis was performed to explore the connection between susceptibility change and microstructure and functional impairment of WD and screen for neuroimaging biomarkers of disease severity.

**Results:**

Wilson’s disease patients demonstrated increased susceptibility in the PU, GP, and TH, and widespread atrophy and microstructural impairments in the PU, GP, CN, and TH. Functional connectivity decreased within the basal ganglia and increased between the PU and cortex. The ROC model showed higher diagnostic value of isotropic volume fraction (ISOVF, in the neurite orientation dispersion and density imaging model) compared with susceptibility. Severity of neurological symptoms was correlated with volume and ISOVF. Susceptibility was positively correlated with ISOVF in GP.

**Conclusion:**

Microstructural impairment of the basal ganglia is related to excessive metal accumulation in WD. Brain atrophy and microstructural impairments are useful neuroimaging biomarkers for the neurological impairment of WD.

## Introduction

Wilson’s disease (WD) is an autosomal recessive inherited disorder of copper metabolism caused by mutations in the ATP7B gene. The ATP7B gene encodes a transmembrane copper-transporting ATPase; the mutation in this gene in WD leads to copper accumulation mainly in the liver and brain (Czlonkowska et al., 2018), resulting in both copper and iron overload (Pak et al., 2021). Previous pathological (Poujois et al., 2017) and quantitative susceptibility mapping (QSM) (Dusek et al., 2021; Ravanfar et al., 2021) studies have demonstrated excessive metal accumulation in the putamen (PU), globus pallidus (GP), caudate nucleus (CN), and thalamus (TH) of WD patients. However, the microstructural and functional alterations of these subcortical nuclei and the diagnostic utility of these measures still remain unclear.

Brain atrophy, microstructural alteration and abnormal functional connectivity of the basal ganglia in WD have been reported in several studies. For example, brain atrophy of the CN, PU, and GP was found to be associated with UWDRS scores (Dusek et al., 2021). With neurite orientation dispersion and density imaging (NODDI), deceased intracellular volume fraction (Vic/ICVF) and increased isotropic volume fraction (Viso/ISOVF) of the basal ganglia (Song et al., 2018) were observed. Functional connectivity between the bilateral CN and fronto-parietal and cerebellar nodes also decreased in neurological WD (Tinaz et al., 2021). However, most of these studies have not comprehensively investigated these impairments and their relationship, or compared their diagnostic value. Meanwhile, pathological studies reported that copper toxicity could cause edema, demyelination and gliosis (Poujois et al., 2017), leading to structural and functional changes in subcortical nuclei. Whether these complicated impairments in WD are related to excessive metal accumulation, which is the crucial pathogenesis of WD, remains unestablished in imaging analysis.

Thus, we performed this multimodal neuroimaging study to: (1) evaluate the metal accumulation in subcortical nuclei of WD patients with QSM; (2) investigate the structural and functional alterations in these nuclei with structural, diffusion and resting-state functional MRI; (3) detect neuroimaging biomarker for diagnosis and monitoring of the disease severity in WD; and (4) explore the relationship between metal accumulation measured by QSM and structural and functional changes. We hypothesized that more severe metal accumulation might be related to more profound structural and functional impairment. This study with multimodal neuroimaging aims to provide novel and comprehensive insights into the mechanism of WD.

## Materials and methods

### Participants

A total of 22 patients diagnosed with Wilson’s disease were enrolled from Beijing Tiantan Hospital, Capital Medical University between May 2021 and March 2022. We also recruited 20 age- and sex-matched healthy controls without any signs or family history of hepatic, neurological or psychiatric disease. This study was approved and supervised by the ethics committee of the Beijing Tiantan Hospital and was performed in accordance with the Declaration of Helsinki. Written informed consents were obtained. Inclusion criteria for WD patients were: (1) diagnosis of WD according to the Leipzig diagnostic criteria (Ferenci et al., 2003) and verified by genetic testing (details of the genetic test were shown in Supplementary material); (2) currently presented with neurological symptoms with Unified Wilson’s Disease Rating Scale (UWDRS) score >0; (3) no history of other significant neurological or psychiatric disorders; and (4) no history of alcohol and other substance abuse which may cause acquired hepatocerebral degeneration. Exclusion criteria: (1) contraindications for MRI including metal implants or fragments and claustrophobia; and (2) psychological disorder, cognitive impairment or involuntary movements causing the participant to be unable to complete the clinical evaluation or MRI scan.

All WD participants underwent clinical evaluation, including laboratory tests (blood routine examination, liver function and serum ceruloplasmin concentration), ophthalmologic examination for Kayser-Fleischer (K-F) ring, genetic testing and structured rating scales. We applied the Global Assessment Scale (GAS) for WD (Aggarwal et al., 2009) to evaluate the global clinical severity of the disease. Neurological symptoms of WD patients were assessed by UWDRS (Członkowska et al., 2007). Cognitive function test was performed using the Mini-Mental State Examination (MMSE).

### Image analysis

The details of image acquisition and image processing are shown in the Supplementary material.

**FIGURE 1:**
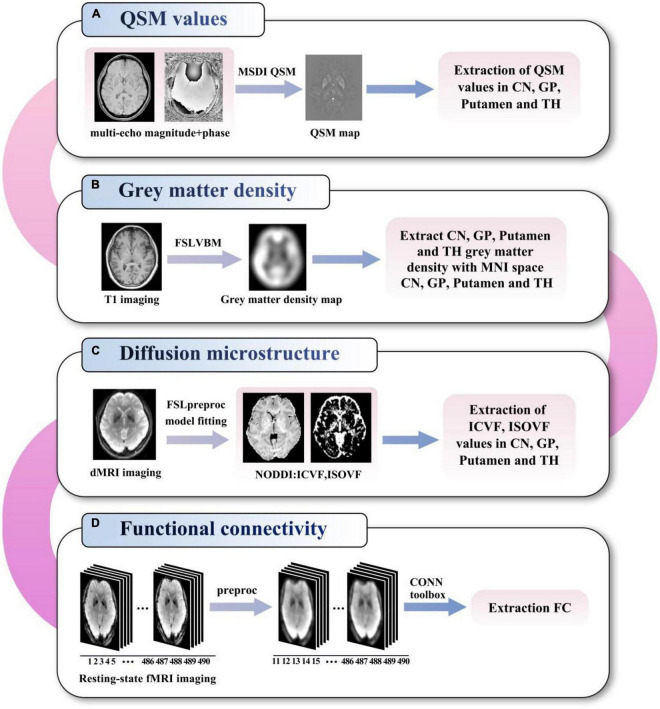
Flowchart of the imaging analysis of QSM values **(A)**, grey matter density **(B)**, diffusion microstructure **(C)**, and functional connectivity **(D)**. QSM, quantitative susceptibility mapping; MSDI, multi-scale dipole inversion; VBM, voxel-based morphometry; MNI, Montreal neurological institute; NODDI, neurite orientation dispersion and density imaging; ICVF, intracellular volume fraction; ISOVF, isotropic volume fraction; FC, functional connectivity; CN, caudate nucleus; GP, globus pallidus; TH, thalamus.

The flowchart of the imaging analysis steps used in this multimodal study is illustrated in Figure 1, and details are provided below. CN, GP, PU and TH ROIs were separately co-registered into different MRI modality (*b* = 0 image in dMRI, magnitude image in QSM, T1 structural MRI) using the CIT168 atlas. In detail, the linear transform with 6 degrees of freedom between T1 structural MRI and specific MRI modality was generated and saved. The non-linear transform between T1 structural MRI and the Montreal Neurological Institute (MNI) 152 template in FSL was generated and saved. The ROIs were determined from the atlas using the concatenations of these transforms.

### QSM images analysis

The multi-echo magnitude and phase images were saved, and QSM was calculated using the multi-scale dipole inversion (MSDI) algorithm (Acosta-Cabronero et al., 2018) by the QSMbox toolbox.^1^ The customized brain mask was extracted from the T1 structural image and linearly transformed into QSM image space. Susceptibility map with the cerebrospinal fluid as the zero reference was used for QSM statistical analysis. The mean magnetic susceptibility values (with the unit of parts per million or ppm) in the CN, GP, PU and TH were analyzed for each participant.

### Gray matter density analysis

The gray matter density (GMD) was calculated with FSL voxel-based morphometry (VBM) (Ashburner and Friston, 2000) pipeline using T1 structural MRI. The smoothed gray matter image in standard space was used for CN, GP, PU, and TH (Forstmann et al., 2014) ROIs gray matter density metric extraction.

### dMRI analysis

For diffusion MRI (dMRI) data, image distortion correction was performed using the TOPUP toolbox (Andersson et al., 2003) in FSL (Smith et al., 2004) using the data collected with reversed-phase-encode blips (AP and PA). The diffusion tensor metrics, including diffusion microstructure metrics ICVF (intracellular volume fraction, termed neurite density index) and ISOVF (isotropic volume fraction) from the NODDI model, were analyzed in the CN, GP, PU, and TH.

### fMRI imaging analysis

Functional MRI (fMRI) data analysis was performed with the Conn toolbox (Whitfield-Gabrieli and Nieto-Castanon, 2012) version 18b and SPM12^2^ running on MATLAB R2020b (MathWorks, Natick, MA, USA). Seed-based functional connectivity analyses were performed at the bilateral CN, GP, PU and TH seeds defined on the mentioned CIT168 atlas.

### Statistical analysis

All statistical analyses were performed using IBM SPSS Statistics (Version 25.0) and R (Version 4.1.2). Differences in gender, age, and clinical data between the WD patients and HCs were examined using chi-squared test and two-sample *t*-test or non-parametric Mann–Whitney test, depending on the data distribution. The ROC curve was applied to evaluate the diagnostic value of the QSM, GMD and ISOVF data using the area under the curve (AUC). Cut-off values of the imaging data were calculated using Youden’s index, which equally weighed sensitivity and specificity. The AUC was compared by DeLong’s test to assess the discriminative power of the ROC models. The mean value of the bilateral nuclei was used in the ROC analysis. Correlation analysis was performed by partial correlation, using age, disease duration and treatment duration as covariates. The significance was set at *P* < 0.05 with FDR correction (to counter the potential bias in multiple comparisons).

## Results

### Participant characteristics

All participants completed all the tests. Detailed demographic and clinical information were shown in Table 1. No significant difference in age and gender was found between the two groups. However, the MMSE in WD group was lower than HC group (*p* = 0.001).

**TABLE 1:** Demographic and clinical characteristics of participants.

Variable	WD (*n* = 22)	HC (*n* = 20)	*P*
Age (year)	29.5 ± 7.8	30.6 ± 6.0	0.599
Gender (male/female)	16/6	15/5	0.867
MMSE	29.0 (28.0, 30.0)	30.0 (29.3, 30.0)	0.001
Disease duration (year)	9.2 ± 7.2	–	–
De-copper treatment duration (year)	7.4 ± 7.2	–	–
GAS for WD	12.1 ± 5.4	–	–
UWDRS	12.5 (7.8, 26.3)	–	–
K-F ring (%)	86.4	–	–

WD, Wilson’s disease; HC, healthy control; MMSE, mini-mental state examination; GAS, global assessment scale; UWDRS, unified Wilson’s disease rating scale; K-F, Kayser-Fleischer.

### Quantitative susceptibility mapping

**FIGURE 2:**
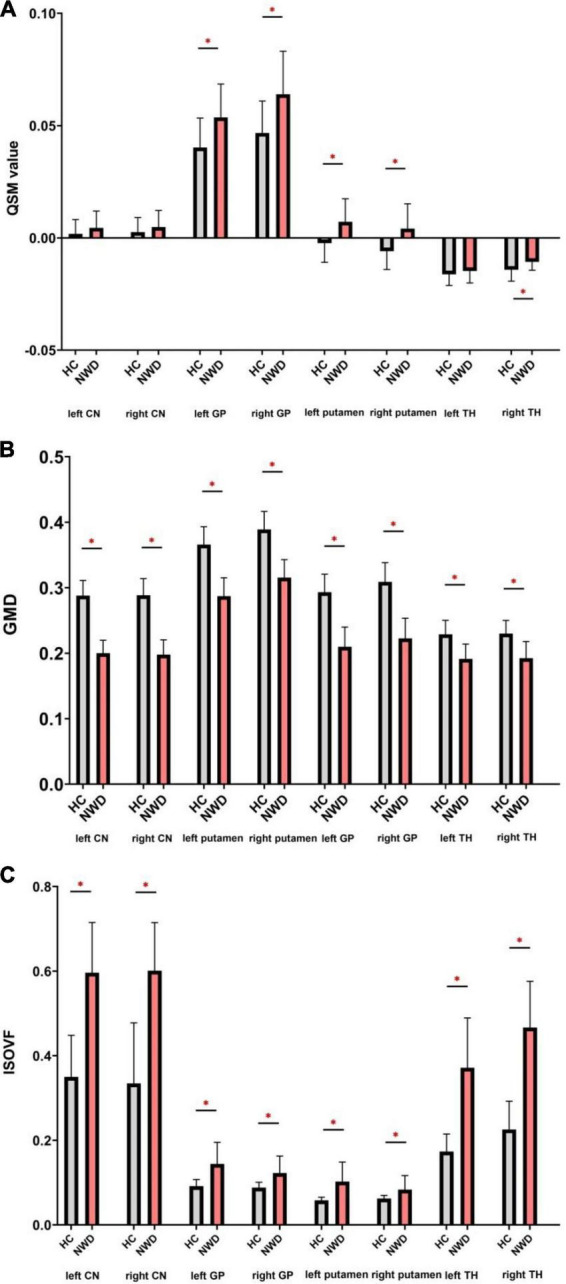
Comparison of the QSM **(A)**, GMD **(B)**, and ISOVF **(C)** values between the WD patients and HCs. QSM, quantitative susceptibility mapping; GMD, gray matter density; ISOVF, isotropic volume fraction; CN, caudate nucleus; GP, globus pallidus; TH, thalamus. *P < 0.05, with FDR correction.

**FIGURE 3:**
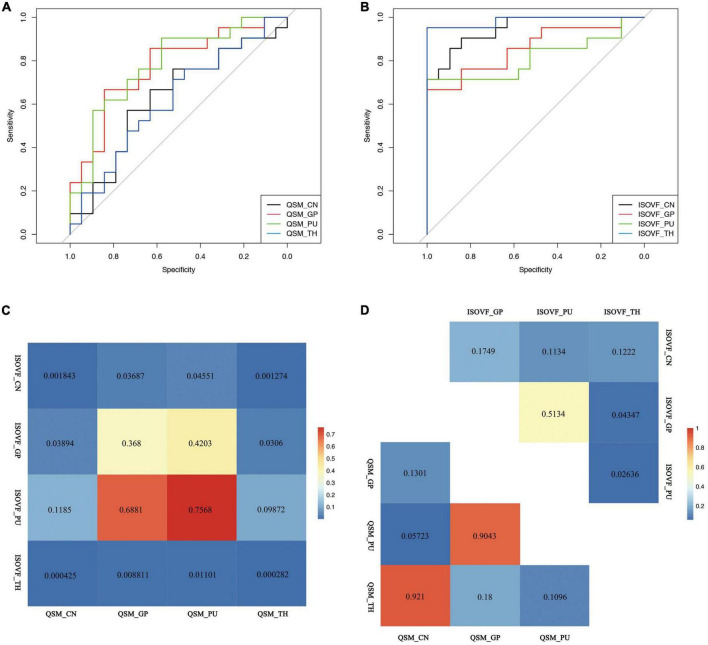
Receiver operating curve (ROC) curve of **(A)** QSM and **(B)** ISOVF value of the CN, GP, PU, and TH and inter-group **(C)** and intra-group **(D)** comparison of the diagnostic value of ISOVF and QSM values. QSM, quantitative susceptibility mapping; ISOVF, isotropic volume fraction; CN, caudate nucleus; GP, globus pallidus; PU, putamen; TH, thalamus.

In WD patients, increased susceptibility was observed in the bilateral PU, bilateral GP and right TH, while the CN and left TH did not show significant difference compared with healthy individuals (Figure 2). The AUC of susceptibility of the CN, GP, PU and TH were 0.63 (cut-off value 0.0044, sensitivity 57, specificity 74%), 0.77 (cut-off value 0.0534, sensitivity 67, specificity 84%), 0.78 (cut-off value −0.0055, sensitivity 90, specificity 58%) and 0.62 (cut-off value −0.0146, sensitivity 71, specificity 53%), respectively, (Supplementary Table 1 and Figure 3A). The DeLong’s test found no significant difference in the AUC of the four nuclei (Figure 3D). The UWDRS score was positively correlated with susceptibility of the right PU (*r* = 0.569, *p* = 0.034).

### Voxel-based morphometry

Wilson’s disease patients showed smaller volumes in the bilateral CN, PU, GP, and TH than HCs (Figure 2). The AUC of GMD of the CN, GP, PU and TH were 0, 0.010, 0.004, and 0.097, respectively (Supplementary Table 1). We established negative correlation between the UWDRS score and volume of the bilateral CN and PU [left CN (*r* = −0.555, *p* = 0.026), right CN (*r* = −0.592, *p* = 0.016), left PU (*r* = −0.597, *p* = 0.030), and right PU (*r* = −0.507, *p* = 0.038)] (Table 2).

**TABLE 2:** Correlation analysis between UWDRS score and GMD and ISOVF values.

		Left CN	Right CN	Left GP	Right GP	Left PU	Right PU	Left TH	Right TH
GMD	r	−0.555	−0.592	−0.293	−0.498	−0.579	−0.507	−0.24	−0.078
	p	0.026	0.016	0.270	0.050	0.030	0.038	0.924	0.760
ISOVF	r	0.558	0.606	0.551	0.571	0.513	0.485	0.585	0.487
	p	0.025	0.017	0.027	0.033	0.050	0.049	0.022	0.056

UWDRS, unified Wilson’s disease rating scale; GMD, gray matter density; ISOVF, isotropic volume fraction; CN, caudate nucleus; GP, globus pallidus; PU, putamen; TH, thalamus.

### Diffusion microstructural imaging

Wilson’s disease patients had significantly higher ISOVF in the bilateral CN, PU, GP, and TH than HCs (Figure 2). ICVF was lower in the bilateral PU but higher in the bilateral GP when comparing WD patients with HCs. ROC analysis showed AUC of 0.95 (cut-off value 0.4709, sensitivity 86, specificity 89%), 0.86 (cut-off value 0.1153, sensitivity 67, specificity 100%), 0.81 (cut-off value 0.0731, sensitivity 71, specificity 100%), and 0.98 (cut-off value 0.2992, sensitivity 95, specificity 100%) at the CN, GP, PU, and TH, respectively (Supplementary Table 1 and Figure 3B). The AUC of TH was significantly higher than the AUC of GP (*p* = 0.043) and PU (*p* = 0.026) (Figure 3D). The UWDRS score was positively correlated with ISOVF of the bilateral CN [left CN (*r* = 0.558, *p* = 0.025), right CN (*r* = 0.606, *p* = 0.017)], bilateral GP [left GP (*r* = 0.551, *p* = 0.027), right GP (*r* = 0.571, *p* = 0.033)], right PU (*r* = 0.458, *p* = 0.049), and left TH (*r* = 0.585, *p* = 0.022). The left PU (*r* = 0.513, *p* = 0.050) and right TH (*r* = 0.487, *p* = 0.056) showed trend of positive correlation though not reaching statistical significance (Table 2).

The AUC of ISOVF of the CN and TH showed better diagnostic value compared with the AUC of QSM of the CN, GP, PU, and TH. The AUC of ISOVF of the GP was significantly higher than the AUC of QSM of the CN and TH (Figure 3C).

### Functional connectivity

**FIGURE 4:**
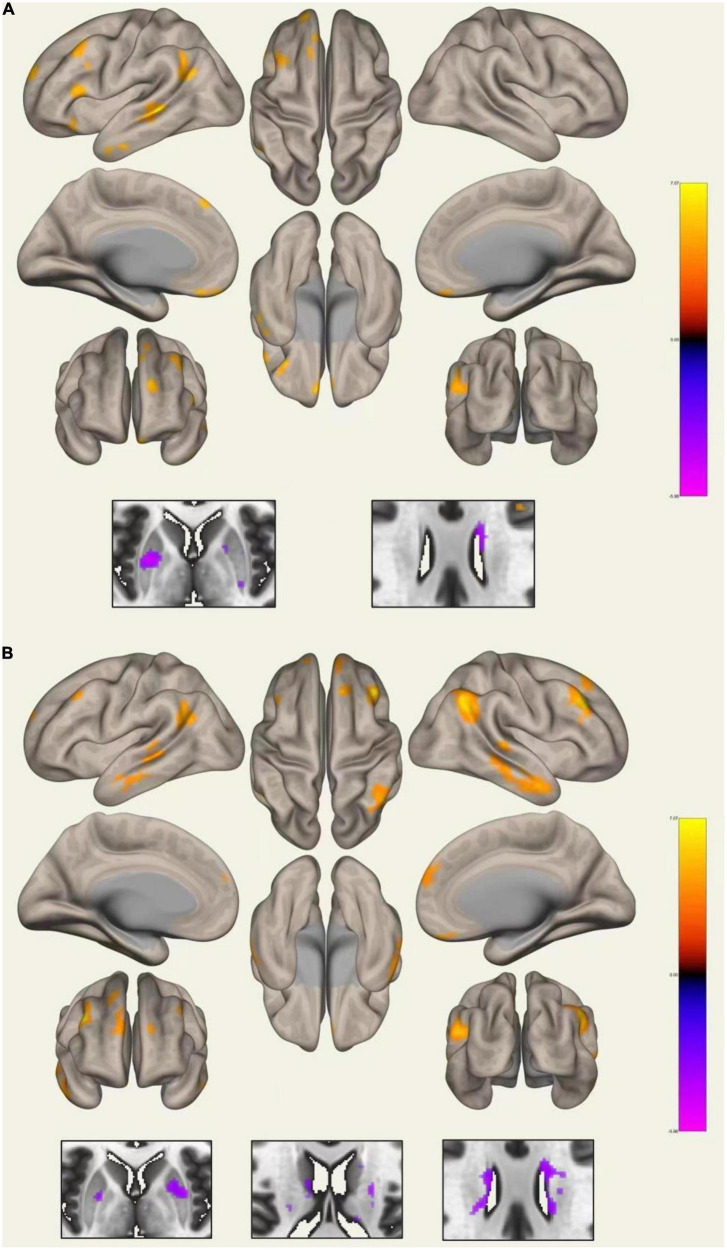
Whole-brain functional connectivity of the left **(A)** and right **(B)** putamen.

Compared with HCs, WD patients had lower FC between the left CN and right CN, and between the right CN and the right PU. Increased FC was detected between the left PU and the left angular gyrus, middle temporal gyrus, middle frontal gyrus, frontal medial cortex, inferior frontal gyrus, frontal pole, frontal orbital cortex, superior frontal gyrus, and decreased connectivity was detected between the left PU and the right PU and left CN. The right PU showed increased connectivity to the right angular gyrus, middle temporal gyrus, middle frontal gyrus, frontal medial cortex, inferior frontal gyrus, frontal orbital cortex, superior frontal gyrus, and decreased connectivity to the left PU and bilateral CN (Figure 4 and Supplementary Table 2). In addition, the WD group showed decreased connectivity between the left GP and the bilateral PU. As for the right GP, decreased connectivity was found between the right GP and the right frontal pole and right PU; increased connectivity was found between the right GP and the left frontal pole. No significant FC change was found for the bilateral TH seed.

### Correlation analysis between QSM and structural and functional measures

Positive correlation was found between QSM values and ISOVF of the bilateral GP in WD (*p* = 0.045). No significant correlation was found between susceptibility and ISOVF of the CN, PU, and TH, and ICVF, GMD, and FC of the CN, PU, GP, and TH.

## Discussion

This study explored the microstructural and functional alterations of subcortical nuclei and their relationship with excessive metal accumulation in WD patients. We observed increased susceptibility at the PU, GP, and TH, widespread atrophy and neurodegeneration at the CN, PU, GP, and TH, decreased connectivity within the basal ganglia and increased connectivity between the PU and cortex in WD patients compared with HCs. The ROC analysis showed higher diagnostic value of ISOVF compared with susceptibility change. Correlation analysis revealed negative correlation between the UWDRS score and volume of the bilateral CN and PU, positive correlation between the UWDRS score and susceptibility of the right PU and ISOVF of the bilateral CN, bilateral GP, right PU, and left TH, and positive correlation between susceptibility and ISOVF of the bilateral GP.

Consistent with previous studies (Yuan et al., 2020; Dusek et al., 2021), we observed increased susceptibility in the bilateral PU, bilateral GP and right TH in WD patients. As for the nature of the susceptibility change of subcortical nuclei in WD patients, both iron and copper accumulation should be taken into consideration. The pathogenesis of WD involves both copper and iron overload (Pak et al., 2021), the majority of intracellular iron is stored as highly paramagnetic ferric iron (Fe3 +) (Dlouhy and Outten, 2013), whereas intracellular copper is stored as Cu + (Scheiber et al., 2017), which is diamagnetic. Previous postmortem MRI and histopathological study of WD patients found correlation between the *R*^2*^ value and iron instead of copper concentration in the PU and GP, and iron concentration was much higher than the copper concentration in the subcortical nuclei of neurological WD patients (Dusek et al., 2017). Thus, the increased susceptibility in the subcortical nuclei might be mainly attributed to excessive iron deposition. However, the effect of copper deposition should also be considered when interpreting the QSM result. The results of CN remained controversial. While several studies (Yuan et al., 2020; Dusek et al., 2021; Jing et al., 2022) reported higher susceptibility of the CN, pathological and seven-tesla MRI studies suggested no difference or only a slight increase of QSM values in CN in neurological WD patients (Fritzsch et al., 2014; Dusek et al., 2017). The inconsistent results might be caused by the variable disease severity and treatment duration of included participants in different studies. Further longitudinal studies with drug-naïve WD patients would help elucidate the QSM change of caudate. While susceptibility change of the basal ganglia was found to aid the diagnosis of WD (Fritzsch et al., 2014), our current results suggest that it is not the measurement with the highest diagnostic efficacy.

In accordance with the previous study (Song et al., 2018), we reported significant atrophy and increased ISOVF of the bilateral CN, PU, GP, and TH in WD patients. Decreased ICVF in the bilateral PU and increased ICVF in the bilateral GP were also found in WD. Brain atrophy was considered to reflect chronic damage (Dusek et al., 2020, 2021) and increased ISOVF might reflect axonal loss and myelin damage in WD. Higher ICVF in the GP suggests increased neurite and neuronal fiber projections in the GP, which may be explained by a compensatory mechanism.

The ROC analysis showed excellent diagnostic accuracy with AUC ≥ 0.9 (Hosmer and Lemeshow, 2000) of the ISOVF of the basal ganglia and TH. Inter- and intra-group comparison showed higher AUC of ISOVF of TH than the GP and PU, and higher AUC of ISOVF than QSM values. The ISOVF proved a more reliable biomarker with high diagnostic value compared with QSM and GMD. After adjusted for age, disease duration and treatment duration, a higher UWDRS score was found to be associated with decreased volume of the bilateral CN and PU, increased susceptibility of the right PU, and increased ISOVF of bilateral CN, GP, right PU, and left TH. Similar result was reported by Dusek et al. (2021) that volume instead of susceptibility of the PU was considered related to neurological severity (Du and Bydder, 2021). A recent longitudinal study evaluating annualized brain atrophy found significantly higher brain atrophy rate in neurological WD and its correlation with changes in functional and neurological severity (Smolinski et al., 2022). Thus, the volumetric change and ISOVF may be considered reliable biomarkers for monitoring chronic impairment in WD. In summary, ISOVF can be considered a promising neuroimaging biomarker for both diagnosis and monitoring of neurological impairment of WD. As researchers believed that the non-significant correlation between susceptibility and UWDRS score might be explained by treatment effect and myelin changes, the ISOVF from NODDI diffusion microstructural model provides a more direct way for evaluation of demyelination, which was found sensitive to copper toxicity (Du and Bydder, 2021).

Functional analysis showed decreased connectivity inside the basal ganglia and increased connectivity between the PU and cortex. These results are similar to those reported by Hu et al. (2019) with decrease functional connectivity strength in the basal ganglia and TH, and increase functional connectivity strength in the dorsolateral prefrontal cortex found in WD patients. Another study demonstrated decreased FC between the basal ganglia and TH, as well as certain regions of the cortex including the cerebellum, cingulate cortex, and superior medial frontal gyrus in WD (Hu et al., 2021), which differs from the findings presented in our study. In a morphometry study measuring both the volume and shape of the subcortical nucleus in neurological WD, researchers found atrophy of the bilateral PU and CN in subregions projecting to the limbic and executive cortex (Zou et al., 2019), which may reflect functional change between the basal ganglia and the cortex. In other movement disorders such as Parkinson’s disease, increased activity of the cerebellar circuits was found to compensate for impairment of the striatum circuits (Appel-Cresswell et al., 2010). Therefore, in this study, the decreased connectivity between the PU, GP, and CN could reflect impairment of the basal ganglia network, whereas the increased connectivity between the PU and cortex might be a compensation for this impairment.

We further explore the relationship between enhanced mental deposition of the subcortical nuclei and other structural and functional measures in WD patients. Positive correlation between susceptibility and ISOVF of the bilateral GP was found, indicating axonal loss and myelin damage could be related to the excessive metal accumulation at GP. Using mice model, Gong et al. (2020) established significant correlation between the density of neurons and neuroglia and susceptibility. No linear relationship was found between QSM values and gray matter density or functional connectivity of subcortical nuclei. As susceptibility change was found to recover after de-copper treatment (Dusek et al., 2021), the ISOVF and nuclei volume may not respond in the same way. In other words, despite recovery of excessive metal accumulation after de-copper treatment, chronic brain atrophy and neurodegeneration may still exist. A recent study found chronic (iron accumulation and brain atrophy) instead of acute (edema, demyelination, and gliosis) brain damage measured by the Dusek scale (Dusek et al., 2020) could predict neurological deterioration in WD (Ziemssen et al., 2022). Therefore, early diagnosis and treatment are important to WD patients. Still, further longitudinal studies with drug-naïve WD patients would be necessary to confirm the results.

This study has certain limitations. First, the number of participants was limited. Second, this is a cross-sectional study with treated participants. Considering the effect of de-copper therapy on metal accumulation, further longitudinal studies with drug-naïve WD patients are needed to validate our results. However, WD is a rare and treatable disease. During the analysis, we have considered the disease and treatment duration as covariates, and our preliminary results did suggest brain impairment in WD patients, consistent with previous studies. Third, the meaning of altered QSM values in WD patients still needs to be further elucidated by pathological studies.

## Conclusion

Overall, this study demonstrated the widespread structural and functional change in the basal ganglia of WD patients. The increased susceptibility, decreased GMD, the neurite microstructural and functional connectivity changes in the basal ganglia may play important roles in the pathogenesis of WD. Microstructure impairment of the basal ganglia, especially ISOVF of the CN and TH, may severe as promising neuroimaging biomarkers for diagnosis and monitoring of the neurological severity of WD. Except for ISOVF of the bilateral GP, no linear relationship was found between susceptibility change and other structural and functional changes. Further longitudinal studies with drug-naïve WD patients would be necessary to confirm the results.

## Data availability statement

The raw data supporting the conclusions of this article will be made available by the authors, without undue reservation.

## Ethics statement

The studies involving human participants were reviewed and approved by the Beijing Tiantan Hospital. The patients/participants provided their written informed consent to participate in this study.

## Author contributions

DS, JJ, and TF contributed to the conception and design of the study. DS, ZhiZ, YG, YZ, XL, JB, LM, HZ, XW, ZW, and HM contributed to the data collection. DS, ZhiZ, ZheZ, SS, JZ, and WL contributed to the data analysis. DS and ZhiZ drafted the manuscript. TW, JJ, and TF performed the review and editing of the manuscript. All authors contributed to the article and approved the submitted version.

## Footnotes

1
https://gitlab.com/acostaj/QSMbox

2
www.fil.ion.ucl.ac.uk/spm/software/spm12/
