# Supplementary material for: Microstructural and functional impairment of the basal ganglia in Wilson’s disease: a multimodal neuroimaging study
Source: Front Neurosci. 2023 Apr 20;17:1146644. doi: 10.3389/fnins.2023.1146644 (PMC10157043; doi:10.3389/fnins.2023.1146644)
Supplement: Supplementary file 1 [file Data_Sheet_1.docx]

**Supplementary materials**

**Supplementary Methods**

**Genetic testing**

A Next Generation Sequencing (NGS) panel related to genetic metabolic diseases was applied to identify the ATP7B gene in the subjects. The chip is customized with a liquid-phase targeted sequence capture system (SureSelect, Agilent), with a total targeted capture region size of 1.32 M. The sequencing data volume is 1.5 G. The data quality control standards are: 1X coverage >99%, 20X coverage >95%, and average sequencing depth of 100X or more.

Briefly, DNA was isolated from peripheral blood and fragmented to build a DNA library using the KAPA Library Preparation Kit (Illumina, Inc., USA). Then, the library was sequenced on the Illumina HiSeq X10 platform (Illumina, San Diego, USA), using a 150-bp paired-end reads according to the standard manual. The sequencing data was filtered with fastp Fq (0.20.0) and aligned with the human reference genome (GRCh37/hg19) by the Burrows-Wheeler Alignment tool (0.7.15-r1140). The samtools (0.1.18-dev) were used to convert the comparison results into bam files. The program picard duplicate (2.17.0) was used to mark the duplicate sequences. After the re-alignment with the human reference genome using GATK (vnightly-2017-03-03-gc6cac2d), we get the final bam files. All the variants on the final bam file were called by haplotypecaller (GATK) first, then were annotated by VEP (105), and were filtered using our own developed program as a final step.

**MRI Data Acquisition**

All participants were scanned on a 3T-MRI scanner (MAGNETOM Prisma, Siemens Healthcare, Erlangen, Germany) using a 64-channel head/neck coil. Three-dimensional (3D) T1 structural MRI, 3D QSM, multishell dMRI and fMRI were acquired for all participants. The 3D T1-weighted images were acquired using sagittal magnetization-prepared rapid gradient echo acquisition (MPRAGE) with the following parameters: TE/TR/TI = 2.0/2000/880 ms, flip angle = 8°, voxel size (AP×RL×SI) = 1×1×1 mm^3^, FOV (AP×RL×SI) = 240×208×256 mm^3^, and acceleration factor = 2. A 3D axial multi-echo gradient-echo (GRE) sequence was used for QSM data acquisition(Liu et al., 2012) with the following parameters: TE1/TE2/TE3/TE4/TR = 6.7/13.4/20.1/26.8/32.0 ms, flip angle = 18°, voxel size (AP×RL×SI) = 0.9×0.9×0.9mm^3^, FOV (AP×RL×SI) = 230×208×158mm^3^, acceleration factor = 4. The 2-shell dMRI images were acquired using the CMRR multiband sequence (version R016a)(Xu et al., 2013) with the following parameters: TE/TR = 70/2800 ms, flip angle (excitation/refocusing) = 78°/160°, voxel size (AP×RL×SI) = 2×2×2 mm^3^, FOV (AP×RL×SI) = 210×210×144 mm^3^, multiband factor =3, diffusion encoding using 50 directions at b = 1000 s/mm^2^ and 50 directions at b = 2000 s/mm^2^, plus 8 (5 AP phase-encoded and 3 PA phase-encoded) additional b = 0 images. The fMRI images were acquired using the CMRR multiband sequence (version R016a) with the following parameters: TE/TR = 39/735 ms, flip angle= 52°, voxel size (AP×RL×SI) = 2.4×2.4×2.4 mm^3^, FOV (AP×RL×SI) = 210×210×154 mm^3^, multiband factor =8, measurements = 490, and the participants were informed to keep their eyes open and to rest during the fMRI scans.

**Image analysis**

**Grey matter density analysis**

The processing follows the FSL VBM(Ashburner and Friston, 2000) pipeline. First, the brain-extracted images were segmented into grey matter, white matter, and CSF. Then, a study-specific grey matter template was created in two steps. The grey matter images were firstly affine-registered to the ICBM-152 grey matter template, and the resulting images were averaged to create a first-pass template. Next, the grey matter images were nonlinearly registered to the first-pass template and then averaged to obtain the final template at 2×2×2 mm^3^ resolution in the standard space. Finally, the grey matter images were nonlinearly registered to the final template and smoothed with a Gaussian kernel with sigma = 8 mm. The voxel values in these images ranged between 0 and 1, representing the percentage of a voxel being grey matter tissue. Regional values of the CN, GP, PU and TH were extracted by averaging the voxel-wise values. Weighted averages of the regions were calculated, with voxels contributing to the average of a region based on their probability of being part of that region.

**dMRI analysis**

Field inhomogeneity-induced distortion, patient motion, and eddy current-related artifacts were corrected using the TOPUP and EDDY toolbox(Andersson and Sotiropoulos, 2016) in FSL. In detail, AP phase-encoded and PA phase-encoded b = 0 images were used to generate the field inhomogeneity map. The field inhomogeneity information and all AP-encoded images (50 directions at b = 1000 s/mm^2^ , 50 directions at b = 2000 s/mm^2^, plus 5 b = 0) were used in EDDY toolbox to correct the image artifacts. The diffusion images after correction were then used for NODDI(Zhang et al., 2012) (using two-shell data of b = 1000 s/mm^2^ , and b = 2000 s/mm^2^ with b = 0) model fitting. The NODDI model fitting used convex optimization for acceleration with AMICO toolbox(Daducci et al., 2015).

**fMRI imaging analysis**

Preprocessing of resting state- (rs-) fMRI images included: removal of the first 10 volumes; realignment; slice-time correction; segmentation of gray matter, white matter, and CSF; normalization to the Montreal Neurological Institute (MNI) template; and spatial smoothing based on a Gaussian kernel set at 6-mm full width at half-maximum. Nuisance variable regression was then performed, and the first 5 principal components from the segmented white matter and CSF were regressed out of the signal.

The 6 motion realignment parameters and their first-order derivatives and outlier volumes detected in the scrubbing procedure were similarly regressed out of the signal. We discarded acquisitions if they had mean framewise displacement values > 0.2 mm or if the maximum displacement was greater than one voxel size (2×2×2 mm^3^). According to these criteria, we excluded 1 WD participant.

**Supplementary Results**

**TABLE S1** ROC model of QSM, GMD, ISOVF in CN, GP, PU and TH

|  | QSM | | | |  | ISOVF | | | |  | GMD |
| --- | --- | --- | --- | --- | --- | --- | --- | --- | --- | --- | --- |
|  | cut-off value | SEN | SPE | AUC |  | cut-off value | SEN | SPE | AUC |  | AUC |
| CN | 0.0044 | 0.57 | 0.74 | 0.63 |  | 0.4709 | 0.86 | 0.89 | 0.95 |  | 0 |
| GP | 0.0534 | 0.67 | 0.84 | 0.77 |  | 0.1153 | 0.67 | 1 | 0.86 |  | 0.010 |
| PU | -0.0055 | 0.90 | 0.58 | 0.78 |  | 0.0731 | 0.71 | 1 | 0.81 |  | 0.004 |
| TH | -0.0146 | 0.71 | 0.53 | 0.62 |  | 0.2992 | 0.95 | 1 | 0.98 |  | 0.097 |

ROC, receiver operating curve; QSM, quantitative susceptibility mapping; GMD, grey matter density; ISOVF, isotropic volume fraction; CN, caudate nucleus; GP, globus pallidus; PU, putamen; TH, thalamus; SEN, sensitivity; SPE, specificity; AUC, area under the curve

**TABLE S2** Clusters with functional connectivity change in WD patients compared to HCs

| **Seed** | **Clusters (x,y,z)** | **brain area** | **size** | **cluster p-FDR** |
| --- | --- | --- | --- | --- |
| left PU | -60, -58, 24 | Angular Gyrus Left | 477 | 0.000001 |
|  | -48, -38, -4 | Middle Temporal Gyrus, posterior division Left | 396 | 0.000002 |
|  | 24, 4, -10 | PU Right | 378 | 0.000003 |
|  | -42, 26, 46 | Middle Frontal Gyrus Left | 278 | 0.000031 |
|  | -6, 50, -22 | Frontal Medial Cortex | 172 | 0.000797 |
|  | -54, 24, 12 | Inferior Frontal Gyrus, pars triangularis Left | 171 | 0.000797 |
|  | -18, 58, 24 | Frontal Pole Left | 166 | 0.000839 |
|  | -38, 32 -20 | Frontal Orbital Cortex Left | 140 | 0.002007 |
|  | -12, 26, 64 | Superior Frontal Gyrus Left | 130 | 0.002687 |
|  | -16, 2, 24 | CN Left | 91 | 0.012825 |
|  | -48, -12, -24 | Middle Temporal Gyrus, anterior division Left | 81 | 0.018647 |
|  |  |  |  |  |
| right PU | 52,-60,38 | Angular Gyrus Right | 1098 | 0 |
|  | 64,-32,-2 | Middle Temporal Gyrus, posterior division Right | 665 | 0 |
|  | -64,-56,18 | Angular Gyrus Left | 632 | 0 |
|  | -24,0,4 | PU Left | 628 | 0 |
|  | 40,30,36 | Middle Frontal Gyrus Right | 586 | 0 |
|  | -70,-30,-4 | Middle Temporal Gyrus, posterior division Left | 326 | 0.000006 |
|  | 12,60,22 | Frontal Pole Right | 321 | 0.000006 |
|  | 22,-20,24 | CN Right | 166 | 0.00078 |
|  | 14,30,52 | Superior Frontal Gyrus Right | 122 | 0.003967 |
|  | -46,-28,-8 | Middle Temporal Gyrus, posterior division Left | 113 | 0.005265 |
|  | 2,48,-28 | Frontal Medial Cortex | 86 | 0.016045 |
|  | -18,-20,24 | CN Left | 70 | 0.032037 |

PU, putamen; CN, caudate nucleus; FDR, false discovery rate

**References**

ANDERSSON, J. L. R. & SOTIROPOULOS, S. N. 2016. An integrated approach to correction for off-resonance effects and subject movement in diffusion MR imaging. *NeuroImage,* 125**,** 1063-1078.

ASHBURNER, J. & FRISTON, K. J. 2000. Voxel-based morphometry--the methods. *NeuroImage,* 11**,** 805-821.

DADUCCI, A., CANALES-RODRíGUEZ, E. J., ZHANG, H., DYRBY, T. B., ALEXANDER, D. C. & THIRAN, J.-P. 2015. Accelerated Microstructure Imaging via Convex Optimization (AMICO) from diffusion MRI data. *NeuroImage,* 105**,** 32-44.

LIU, T., XU, W., SPINCEMAILLE, P., AVESTIMEHR, A. S. & WANG, Y. 2012. Accuracy of the morphology enabled dipole inversion (MEDI) algorithm for quantitative susceptibility mapping in MRI. *IEEE Transactions On Medical Imaging,* 31**,** 816-824.

XU, J., MOELLER, S., AUERBACH, E. J., STRUPP, J., SMITH, S. M., FEINBERG, D. A., YACOUB, E. & UĞURBIL, K. 2013. Evaluation of slice accelerations using multiband echo planar imaging at 3 T. *NeuroImage,* 83.

ZHANG, H., SCHNEIDER, T., WHEELER-KINGSHOTT, C. A. & ALEXANDER, D. C. 2012. NODDI: practical in vivo neurite orientation dispersion and density imaging of the human brain. *Neuroimage,* 61**,** 1000-16.
